# Development of the Protective Strategies for Psychedelics Scale: A novel inventory to assess safety strategies in the context of psychedelics

**DOI:** 10.1177/02698811231214060

**Published:** 2023-12-04

**Authors:** Maha N Mian, Brianna R Altman, Fiona Low, Mitch Earleywine

**Affiliations:** 1 Department of Psychiatry and Behavioral Sciences, Weill Institute for Neurosciences, University of California, San Francisco, CA, USA; 2 Division of Research, Kaiser Permanente Northern California, Oakland, CA, USA; 3 Department of Psychology, Rutgers University, New Brunswick, NJ, USA; 4 University at Albany, SUNY, Albany, NY, USA

**Keywords:** Protective behavior strategies, harm reduction, psychedelics, safety, hallucinogens

## Abstract

**Background:**

Individuals who use psychedelics take efforts to mitigate unintended consequences. Despite the demonstrated utility of analogous protective behavioral strategies (PBS) assessments for other substances, no standardized scale exists to capture these protective strategies for psychedelic use.

**Objective:**

The present study addresses a notable gap concerning the assessment of psychedelic use, specifically by developing a scale measuring the protective strategies employed around use, called the Protective Strategies for Psychedelics Scale (PSPS).

**Methods:**

A sample (*M*_age_ = 36.85 years old, standard deviation = 10.3; male = 61.9%; White = 85.2%) of 434 adults with lifetime use of psychedelics reported on initial qualitatively developed items for the PSPS, PBS scales for cannabis and alcohol, and use of alcohol, cannabis, and psychedelics.

**Results:**

Iterative principal components analyses began with 37 items and yielded a 32-item two-factor solution demonstrating excellent internal reliability (Cronbach’s α = .95) and accounted for 51.3% of the variance. Nineteen items loaded on PSPS factor 1, which focused on long-term preparation, emphasizing strategies focused on mood/intentions, preparing the substance, environment, and scheduling episode of use; 13 items loaded on factor 2, which focused on short-term preparation, highlighting strategies surrounding social context, health, and other substances. The PSPS demonstrated convergent validity with validated PBS scales for cannabis and alcohol (*r* = 0.71–0.79, *p* < 0.001), and was moderately associated with lifetime psychedelic use (*r* = 0.28, *p* < 0.001).

**Conclusion:**

The PSPS demonstrates promising psychometric properties, and future work validating the scale for diverse samples across research and clinical settings is warranted.

## Introduction

Psychedelic use has been long documented for both recreational and spiritual purposes. Renewed interest in their therapeutic application stems from documented impact on mental health. For example, psilocybin, ayahuasca, and 3,4-methylenedioxymethamphetamine (MDMA) mitigate symptoms of anxiety, depression, and addiction (Bogenschutz et al., 2015; Carhart-Harris and Goodwin, 2017; Garcia-Romeu et al., 2019; Griffiths et al., 2016; Sanches et al., 2016). Ketamine alleviates depression (Sumner et al., 2021), and lysergic acid diethylamide (LSD) in combination with therapy alleviates anxiety (Gasser et al., 2014; Holze et al., 2023). Emerging work on ibogaine and peyote also suggests they can address substance use concerns, while naturalistic mescaline can improve various mental health problems (Agin-Liebes et al., 2021; Winkelman, 2015). Such work demonstrates the therapeutic potential of psychedelics, spurring renewed efforts to determine their efficacy.

Meanwhile, many individuals in the community continue to use psychedelics for recreation and relief (Yockey and King, 2021). Many people using psychedelics take efforts to mitigate unintended consequences and heighten their anticipated experience using strategies before, during, and after psychedelic use (Fernández-Calderón et al., 2019; Hupli et al., 2019; Lancelotta and Davis, 2020a; Lea et al., 2020; Ruane, 2017). Given the diversity of substances, many grassroots-developed strategies exist, with common themes. For instance, individuals might receive support from others with experience using psychedelics (Lancelotta and Davis, 2020b). This approach can include collecting information from experienced guides prior, being guided through, or debriefing after (Johnson et al., 2008). Other strategies involve planning when and where to use, predetermining dosage, monitoring subjective effects during use, and refraining from co-using psychedelics with other substances, such as alcohol, depressants, or stimulants (Fernández-Calderón et al., 2019; Lancelotta and Davis, 2020a). These techniques have great potential to increase safety like comparable protective behavioral strategies (PBS), which specifically reduce negative consequences from use.

PBS come from a broader harm reduction framework. PBS scales for alcohol and cannabis have informed intervention efforts, increased safety behaviors, and reduced risk of adverse outcomes (Martens et al., 2005; Pedersen et al., 2016; Prince et al., 2013, 2019). Existing reports of analogous strategies surrounding psychedelic use (e.g., monitoring consumption and quantity) appear to reduce harm and increase enhancement but lack standardization (Hupli et al., 2019; Lancelotta and Davis, 2020a; Lea et al., 2020; Ruane, 2017). As a result, the prevalence of PBS for psychedelics is unclear, and the dissemination of these strategies in community settings or around personal psychedelic use might be limited.

Standard assessment of PBS for psychedelics requires a psychometrically validated scale. Previous studies indicate that harm reduction is relevant for psychedelic use (Hupli et al., 2019; Lancelotta and Davis, 2020a; Lea et al., 2020; Ruane, 2017), and even develop their own items to assess protective strategies (Fernández-Calderón et al., 2019; Lancelotta and Davis, 2020a). While these methods offer important contributions, this construct requires a psychometrically validated measure. The development of an accurate and validated measure is essential for expanding the research of any construct (Noar, 2003). Such a measure would also assist in examining correlates of PBS with relevant outcomes (e.g., mystical experiences, therapeutic impact). PBS for alcohol and cannabis demonstrate trends such that individuals who use more protective strategies appear buffered from harms related to their substance use (Araas and Adams, 2008; Prince et al., 2013). PBS might buffer problems related to psychedelic use as well (Hupli et al., 2019; Lea et al., 2020; Ruane, 2017). Importantly, psychedelics differ from cannabis and alcohol in subjective effects (Carhart-Harris et al., 2018; Haijen et al., 2018). For example, alcohol and to a certain extent, cannabis, have depressant effects; highly potent cannabis products might prompt hallucinogenic type effects, but often with adverse components (Earleywine et al., 2021; Farmer et al., 2019). While risk mitigation is a primary focus for alcohol and cannabis, individuals who use psychedelics look to enhance subjective effects (e.g., mysticism, insight, integration) (Lancelotta and Davis, 2020b). PBS for other substances simply might not apply to psychedelics.

The present study addresses a notable gap concerning the assessment of psychedelic use by developing a scale measuring the protective strategies. With items generated through qualitative methods, we aimed to develop an original scale capturing protective strategies for psychedelics. We chose to reframe the construct as protective strategies given the potential for cognitive and affective strategies as well as behavioral ones, and named the inventory as the “Protective Strategies for Psychedelics (PSPS)” scale. We also examined links with other validated PBS scales. We hypothesized that a principal components analysis (PCA) will yield a reliable solution. Given the novelty of this scale, we did not have specific hypotheses about what dimensions might arise. We also hypothesized that the scale generated from this analysis will demonstrate validity related to other PBS scales (PBSS, Protective Behavioral Strategies for Marijuana (PBSM); Martens et al., 2005; Pedersen et al., 2016) and inversely covary with alcohol, cannabis, and psychedelic use, consistent with protective strategies (Araas and Adams, 2008; Fernández-Calderón et al., 2019; Pearson et al., 2019; Pedersen et al., 2017).

## Methods

### Participants and procedures

Participants were recruited through Amazon’s Mechanical Turk (MTurk), where they viewed the study description and eligibility requirements. MTurk is a crowdsourcing task-oriented platform. Interested individuals can volunteer to participate in a study in exchange for monetary reward. MTurk study recruitment has advantages over other recruitment methods, including drawing a sample from a diverse, nationally representative population and engaging individuals who use substances that typically have lower base rates in the community (Strickland and Stoops, 2019). We incorporated best practices for MTurk data collection, including quality checks, transparent exclusion and qualification criteria, and minimum wage compensation (Mellis and Bickel, 2020). Participants who opted to participate were directed to the study site hosted on Qualtrics. Participants were presented with an informed consent form, outlining the study purpose, eligibility criteria, risks and benefits to participating, and contact information for further inquiries and additional resources. Eligible, consented participants who passed quality checks and completed the survey were rewarded for participation ($2.40). All procedures were in accordance with and approved by the university Institutional Review Board (IRB).

### Measures

#### Screening and demographics

Participants were presented with initial screening questions to determine eligibility, including age (18 years and older), psychedelic use (any lifetime use, currently using), and residency in the United States (determined through MTurk). Psychedelic use was screened with the following item: “Have you ever used hallucinogens or psychedelic drugs (e.g., LSD, psilocybin mushrooms, psilocybin, DMT, ayahuasca, salvia divinorum, peyote, mescaline, PCP, laudanum, MDMA (X, ecstasy), ketamine (K, Vitamin K), DXM (robed, robo-tripping), ibogaine, DOT (aleph))?” We intentionally sought to recruit individuals who use different kinds of psychedelics to better capture the variability of using different protective strategies. Participants also reported on sex, gender, ethnicity, race, and level of education.

#### Development of the PSPS

We initially sought to explore experiences of individuals using psychedelics through a qualitative study, also approved by the university IRB. In this preliminary work, we recruited 148 respondents (75.3% male; *M*_age_ = 28.8) who use psychedelics through social media postings (e.g., Facebook, Reddit). Participants provided open-ended responses to prompts about their use, negative and positive consequences following use, and strategies they use either before, during, or after their use (Low et al., 2021). Most participants used some form of protective strategies (80%), and thematic coding and content analysis revealed several relevant themes and generated 37 specific items. Common strategies included preparing the environment one used psychedelics in, cultivating a specific mindset prior to use, and managing personal and professional responsibilities around a planned episode to use. Members of the research team reviewed items for consistency, readability, and redundancy. We chose an item stem and response scale consistent with previously validated PBS scales (Martens et al., 2005; Pedersen et al., 2016): “Please indicate the degree to which you engage in the following behaviors when using psychedelics” followed by: 1 (*never*) to 6 (*always*). Scale reliability is discussed in results.

#### Psychedelic use

Participants reported on lifetime use for the following substances: LSD (acid); Psilocybin Mushrooms (‘shrooms); Psilocybin (pure form); DMT (other than ayahuasca); Salvia divinorum; Peyote; Mescaline (other than peyote); PCP (Phencyclidine); Laudanum; MDMA (3-4 methylenedioxymethamphetamine; X, ecstasy); Ketamine (K, Vitamin K); Ibogaine; DOT (2,5-dimethoxy-4-methylthioamphetamine; aleph); Ayahuasca. Participants also responded to question regarding co-use with other substances (“When you use psychedelics, do you use psychedelics with other substances or alone?”), awareness of what psychedelic one used (“Are you always aware of what substance you are using when you use psychedelics?”), frequency of use in the past 5 years (“In the last 5 years, how often did you engage in psychedelic use?”), frequency of use in the past week, and rated their worst psychedelic experience (−50 (“extremely negative”) to 50 (“extremely positive”)). We also assessed motivation for use (“Which of the following best describes your typical motivation for using psychedelics?”) with response items: religious/spiritual use; therapeutic use; recreational use; adapted from (Uthaug et al., 2021) to better understand in what contexts individuals might be employing protective strategies for psychedelics.

#### Other substance use

Cannabis and alcohol use were also assessed through past week frequency and weekly quantity (cannabis responses: less 1/8th g, 1/8th g, 1/4th g, ½ g, 3/4th g, 1 g, more than 1 g).

#### Other PBS scales

Participants who reported cannabis use and/or alcohol use also completed the PBSM Scale and the Protective Behavioral Strategies Survey (PBSS), respectively (Martens et al., 2005; Pedersen et al., 2016). Participants rated each item from 1 (*never*) to 6 (*always*). Both scales demonstrated good internal reliability in our sample (PBSM Cronbach’s α = .93, PBSS Cronbach’s α = .91).

### Data analytic plan

All analyses were conducted using SPSS 28. A total of 434 eligible adults completed the survey passing all quality checks. Forty participants were removed for not indicating lifetime use of psychedelics and three were removed for no response, leaving a final sample of 391. Less than 3% of data were missing, allowing for multiple imputation of missing values. Data were examined for violations of normality, homoscedasticity, and multicollinearity based on scatterplots and bivariate correlations. Assumptions for PCA were also met and are detailed below. Three variables were skewed (cannabis frequency = 1.8, lifetime psychedelic use = 8.6, alcohol quantity = 4.7); Box–Cox transformations were applied and brought skews to an acceptable level (cannabis frequency = −0.34, lifetime psychedelic use = −0.20, alcohol quantity = −0.13). All subsequent analyses used the transformed variables; untransformed values are reported unless otherwise specified (Osborne, 2013).

Initial descriptive analyses examined demographic frequencies, means and standard deviations for substance use measures and validated scales (PBSM, PBSS). We conducted a PCA to examine the underlying factor structure of the rated protective strategies items. PCA is an appropriate choice to explore potential patterns in our data without preconceived theoretical constructs, and optimally reduce the number of overall items while still retaining the most variance; this procedure is also consistent with the scale development of the PBSM and PBSS (Pedersen et al., 2016; Tabachnick & Fidell, 2013). An initial PCA allowed us to examine the factor loadings for each proposed item, and subsequently remove items with low loadings (<0.40). We conducted iterative PCAs to produce a final set of items. Dimensionality was indicated through patterns demonstrated by factor loadings and corroborated with scree plots and parallel analysis (Horn, 1965). Oblique rotation was applied as extracted components appear moderately associated (*r* > 0.32; (Tabachnick & Fidell, 2013). Inter-item correlation coefficients above 0.90 and low communalities indicated potential redundant or poor-fitting items that were removed from the final scale.

We examined the internal reliability of the overall scale and individual components using Cronbach’s α. We also examined alpha-if-deleted values to determine if removal of individual items improved internal reliability. Finally, criterion validity of the generated scale was determined through bivariate associations between the final scale and validated PBS scales (PBSS, PBSM) and substance use (psychedelics, alcohol, cannabis).

## Results

### Sample

Complete sample descriptives can be found in Table 1. On average, participants were 36.85 years old (standard deviation (SD) = 10.3). The majority of the sample identified as male for both sex (61.9%) and gender identity (61.1%). The sample was primarily White (85.2%) and 24.6% identified as having Hispanic/Latin/Spanish origin. Participants were also highly educated, as 61.1% reported having a bachelor’s degree and 25.8% reported completing a master’s degree.

**Table 1.** Sample descriptives (*N* = 434).

	%
Sex	
Male	61.9
Female	37.6
Gender	
Male	61.6
Female	38.1
Nonbinary	0.3
Race	
American Indian or Alaska Native	1.0
Asian or Asian-American	2.0
Black or African American	7.4
Hispanic or Latinx	2.8
White	85.2
Multiracial	1.3
Education	
Some high school	0.5
High school/General Educational Development	6.4
Some college	2.8
Associates	3.1
Bachelors	61.1
Some graduate training	25.8
Doctoral degree	0.3
Past 5 year psychedelic use	
Daily	12.3
Weekly	36.3
Monthly	23.8
A few times per year	18.7
Yearly	4.6
Less than yearly	2.0
Never	1.3
Psychedelic co-use	
By themselves	92.6
With other substances	6.1
Motivation for psychedelic use	
Religious/spiritual use	36.6
Therapeutic use	43.5
Recreational use	19.9
Cannabis quantity	
Less than 1/8th g	14.8
1/8th g	19.4
1/4th g	15.6
½ g	11.3
¾ g	6.9
1 g	5.9
More than 1 g	8.7

### Substance use

Regarding psychedelic use history, participants used an average of nearly 50 times in their lifetime (*M* = 49.9, SD = 82.4, range 1–1052) and the vast majority of participants indicated awareness of what psychedelics they used (95.7%). Mescaline was most frequently used on average (*M* = 9.05, SD = 67.7), while psilocybin mushrooms were endorsed by the most participants (*n* = 327, 75.3%); further details regarding lifetime use of each psychedelic and endorsement by sample can be found in Table 2. Lifetime use of more than one psychedelic was common; 363 individuals reported using at least two different psychedelics in their lifetime, and the average number of different psychedelics used was 8.38. In the past 5 years, over a third of the sample reported using psychedelics on a weekly basis (36.3%), followed by monthly use (23.8%), a few times per year (18.7%), daily use (12.3%), and yearly use (4.6%). On average, participants used psychedelics about 3 times per week (*M* = 3.17, SD = 3.5). Psychedelics were typically taken alone (92.6%) and primarily for therapeutic use (43.5%), followed by religious/spiritual reasons (36.6%) and recreation (19.9%). On average participants reported positive experiences with psychedelics (*M* = 19.6, SD = 21.7; *range* −50–50).

**Table 2.** Lifetime psychedelic use (*N* = 434).

Psychedelic	Lifetime use *M* (SD)	Sample endorsing any lifetime use *n* (%)
LSD	5.44 (9.35)	259 (59.7%)
Psilocybin shrooms	7.25 (13.5)	327 (75.3%)
Psilocybin pure	4.62 (5.69)	253 (58.3%)
DMT	4.41 (4.19)	237 (54.6%)
Salvia	4.77 (7.79)	235 (54.1%)
Peyote	5.13 (9.62)	228 (52.5%)
Mescaline	9.05 (67.7)	218 (50.2%)
PCP	4.22 (3.49)	214 (49.3%)
Laudanum	4.23 (3.57)	199 (45.9%)
MDMA	7.58 (32.7)	241(55.5%)
Ketamine	6.00 (16.1)	239 (55.1%)
Ibogaine	4.47 (4.76)	191 (44.0%)
DOT	3.90 (3.31)	179 (41.2%)
Ayahuasca	4.07 (3.97)	174 (40.1%)
Other	7.68 (21.7)	65 (14.9%)

*M* = mean; SD: standard deviation; LSD: lysergic acid diethylamide.

Most of the sample endorsed lifetime use of cannabis (82.9%) and alcohol (90.8%). Participants used cannabis about 12 times per week (*M* = 12.4, SD = 11.7), with 74% using about half a gram or less per week. On average, participants consumed alcohol about three times per week (*M* = 3.64, SD = 1.9) and had almost four drinks per week (*M* = 3.94, SD = 3.9). The sample moderately endorsed the use of both cannabis and alcohol PBS (PBSM *M* = 72.4, SD = 15.2; PBSS = 64.5, SD = 11.6; range 0–100).

### Protective strategies for psychedelics scale development

Initial extraction was a PCA of the 37 items generated from preliminary qualitative analyses. Assumptions for analysis were met and indicated adequate factorability; the Kaiser–Meyer–Olkin (KMO) measure of sampling adequacy was 0.958, the Bartlett’s test of sphericity was significant (χ^2^ = 8779.76, df = 666, *p* < 0.001), and all communalities were of moderate strength (>0.4, 0.486–0.759). Five factors were extracted with eigenvalue greater than or equal to 1 (15.55, 3.09, 1.42, 1.19, 1.16). Visual examination of the scree plot and comparison with a parallel analysis supported a two-factor solution (Hayton et al., 2004; Horn, 1965). The variance accounted by the initial eigenvalues also supported a two-factor solution, as the eigenvalue of 15.55 accounted for 42.01% of the variance, followed by 3.09 accounting for 8.35% of the variance, while the remaining factors contribute less than 4% of the variance each.

In our second extraction, we re-specified the PCA to extract a two-factor solution. Once again the KMO and Bartlett’s test demonstrated that the data were factorable (KMO = .958; Bartlett’s: χ^2^ = 8779.76, df = 666, *p* < 0.001), and all communalities remained greater than .4. A two-factor model (eigenvalues = 15.55, 3.09) accounted for 50.36% of the variance. Five items (8, 12, 17, 21, and 28) were cross-loaded on both factors and subsequently removed (Table 3). The strong associations between factors (*r* = 0.55) indicated the application of a promax (oblique) rotation.

**Table 3.** Protective strategies for psychedelics scale PCA.

	Mean (SD)	% endorsed	Factor loadings	Communalities (*h*^2^)
*Items removed due to overlapping loadings in two-factor solution PCA*				
8. I get plenty of rest before I use one of these substances.	4.28 (1.12)	98.7	0.436, 0.313	
12. I clean the space I will be using before taking these substances.	4.36 (1.12)	99.0	0.457, 0.308	
17. I make sure I have used the bathroom before I use these substances.	4.22 (1.25)	96.7	0.344, 0.454	
21. I prepare activities/items I might enjoy while using the substance (e.g., music playlists, hobby supplies, books, specific snacks).	4.28 (1.13)	98.2	0.445, 0.370	
28. I avoid using other substances (e.g., alcohol, tobacco, marijuana, other illicit substances) entirely during my use of these substances.	4.18 (1.29)	96.9	0.393, 0.314	
*Factor 1: Mood/intention, substance, environment, scheduling*				
1. I make sure that I am in the right mood before I use these substances.	4.42 (1.07)	99.5	0.762	0.528
3. I set an intention for using one of these substances.	4.42 (1.12)	98.5	0.513	0.425
4. I think very carefully about what I want to get out of the experience before I use these substances.	4.32 (1.13)	98.5	0.503	0.465
6. I remind myself not to fight the experience or be alarmed before I use these substances.	4.25 (1.21)	97.2	0.473	0.423
7. I avoid using these substances when I am experiencing negative or unstable mood states.	4.38 (1.18)	98.7	0.685	0.474
9. I am careful about what I have scheduled the next day when I use one of these substances.	4.49 (1.18)	98.7	0.731	0.560
10. I take care of obligations or loose ends before I use these substances.	4.38 (1.21)	98.5	0.706	0.485
11. I would alter my plans to use these substances if something unexpected came up on the day I planned to use them.	4.32 (1.20)	98.5	0.533	0.399
16. I make sure I have adequate water and anything else I might want in my stomach before I take one of these substances.	4.45 (1.21)	97.4	0.741	0.560
18. I make sure that I have whatever I might need before I take one of these substances.	4.49 (1.18)	98.5	0.707	0.582
19. I make sure that I am in a comfortable environment before I take one of these substances.	4.54 (1.16)	99.0	0.841	0.588
22. I make sure I am dressed comfortably before using these substances.	4.35 (1.17)	98.7	0.568	0.464
23. I would avoid using these substances if I felt I was in an unsafe environment (e.g., with people I did not know, in an unfamiliar space).	4.48 (1.15)	99.5	0.889	0.640
24. I make sure that I am around the right people before I take one of these substances.	4.46 (1.16)	98.7	0.785	0.536
29. I only get these substances from a reliable source.	4.50 (1.09)	99.5	0.847	0.593
33. I make sure I know how I will travel/get home while I am using these substances.	4.48 (1.17)	98.5	0.714	0.488
34. I plan out where I will be and what I will do during my trip before I use these substances.	4.44 (1.14)	98.5	0.673	0.537
36. I do research before using these substances to know the right dosage, what to expect, and any precautions I should take.	4.29 (1.22)	98.0	0.525	0.407
37. I am careful about my dosage.	4.78 (1.14)	99.0	0.705	0.448
*% Variance*	*41.73*			
*Factor 2: Social, health, and other substances*				
2. I pray, meditate, or relax before I use these substances.	4.17 (1.33)	95.5	0.614	0.465
5. I talk about what I want to get out of the experience before I use one of these substances.	4.16 (1.18)	96.7	0.683	0.545
13. I make significant lifestyle changes (e.g., changing my diet, refraining from other substance use, refraining from sexual activity, taking supplements, fasting) in the days before I use these substances.	4.14 (1.26)	96.2	0.754	0.513
14. I watch what I eat before I use one of these substances.	4.18 (1.19)	96.9	0.616	0.519
15. I exercise, stretch, or do some form of physical activity before taking these substances.	4.04 (1.35)	94.9	0.795	0.617
20. I make sure my phone is off before I use one of these substances.	4.02 (1.34)	93.9	0.776	0.550
25. I discuss rules and expectations for the trip with others who will be using the substance with me.	4.25 (1.25)	96.9	0.523	0.491
26. I prepare additional doses of the substance in advance if I intend to consume more halfway through the day.	4.10 (1.26)	96.4	0.792	0.548
27. I prepare other substances such as alcohol, tobacco or marijuana to be used in conjunction with the substance.	3.99 (1.31)	93.9	0.741	0.422
30. I have a designated “sitter” when I use these substances.	4.05 (1.28)	96.4	0.754	0.552
31. I have experienced users around when I use these substances.	4.31 (1.18)	97.2	0.562	0.467
32. I make sure to inform someone about where I will be and my intention to use these substances.	4.16 (1.25)	96.4	0.598	0.580
35. I test the substances for adulterants before using them.	4.07 (1.31)	93.6	0.829	0.554
*% Variance*	*9.60*			

Stem for each item: “Please indicate the degree to which you engage in the following behaviors when using psychedelics”; response options: 1 (*never*) to 6 (*always*).

PCA: principal components analysis; SD: standard deviation.

In the final extraction, we respecified the PCA to extract the two-factor solution with a promax rotation among the 32 remaining items (KMO = 0.955; Bartlett’s: χ^2^ = 7311.97, df = 496, *p* < 0.001). The final two-factor solution comprised of 19 items loaded onto factor 1 (eigenvalue = 13.35) accounting for 41.73% of the variance, and 13 items on factor 2 (eigenvalue = 3.07), accounting for 9.60% of the variance (Table 3). The overall PSPS scale demonstrated excellent internal reliability (Cronbach’s α = 0.95), and alpha-if-deleted values suggested that reliability could not be improved with the removal of any items. Averages for all items ranged between 3.99 and 4.78, and all items were frequently endorsed. PSPS factor 1 focused on long-term preparation, emphasizing strategies focused on mood/intentions, preparing the substance, environment, and scheduling episode of use, and demonstrated excellent reliability (Cronbach’s α = 0.94). PSPS Factor 2 focused on short-term preparation, highlighting protective strategies surrounding social context, health, and other substances and also had good reliability (Cronbach’s α = 0.92). Alpha-if-deleted values for both factors indicated that reliability could not be improved with item removal.

### PSPS validity

We examined the PSPS scale and the two factors in relation to previously validated PBS scales to determine convergent validity, and to substance use indices to determine criterion validity. All reported correlations were calculated with transformed values. A global PSPS score was calculated by summing all 32 items. The global PSPS was correlated with cannabis PBS (PBSM; *r* = 0.71, *p* < 0.001) and alcohol PBS (PBSS; *r* = 0.79, *p* < 0.001). Both PSPS factors also associated with both cannabis PBS (*r*’s = 0.64–0.65, *p* < 0.01) and alcohol PBS (*r*’s = 0.70–0.71, *p* < 0.01). The global PSPS score was also moderately associated with lifetime psychedelic use (*r* = 0.28, *p* < 0.001), and weakly associated with cannabis frequency (*r* = 0.12, *p* < 0.05) and alcohol frequency and quantity (*r* = 0.13, *r* = 0.12, respectively, *p* < 0.05). Notably, lifetime psychedelic use was also positively associated with cannabis PBS (*r* = 0.17, *p* < 0.001). The average for the total PSPS was 137.90 (SD = 24.79, *range* 32–192), and average for factor 1 was 53.64 (SD = 11.81, *range* 13–78) and average for factor 2 was 84.26 (SD = 15.51, *range* 19–114.80) (see Table 4 for correlations).

**Table 4.** Bivariate associations.

	1	2	3	4	5	6	7	8
1. PSPS								
2. PSPS factor 1	0.931**							
3. PSPS factor 2	0.877**	0.640**						
4. PBSM	0.711**	0.652**	0.636**					
5. PBSS	0.789**	0.714**	0.701**	0.725**				
6. Psych lifetime	0.280**	0.302**	0.190**	0.169*	0.101			
7. Alc frequency	0.129*	−0.06	0.340**	0.131*	0.019	0.267**		
8. Alc quantity	0.112*	−0.056	0.303**	0.081	0.007	0.217**	0.617**	
9. Cannabis frequency	0.124*	0.002	0.252**	−0.066	0.098	0.417**	0.488**	0.356**

Correlations calculated from transformed values.

PSPS: Protective Strategies for Psychedelics Scale; PBSM: Protective Behavioral Strategies for Marijuana; PBSS: Protective Behavioral Strategies Survey; Psych lifetime: lifetime use of psychedelics; Alc: alcohol. Bold indicates significant values.

* *p* < 0.05. **p < 0.001.

## Discussion

The objective of the present study was to develop a novel scale capturing protective strategies used in the context of psychedelics. At present, no psychometrically validated measure for protective strategies for psychedelics use exists. We sampled community members reporting lifetime psychedelic use experience to develop and validate a novel 32-item scale, the Protective Strategies for Psychedelics Scale (PSPS). The PSPS comprises of two subfactors that capture both long- and short-term harm reduction strategies that individuals might employ to mitigate potential problems or unintended consequences related to use. The global scale accounted for 51.33% of the variance, and both the global scale and individual factors demonstrated excellent internal consistency. These results are comparable to the development of previously validated PBS measures for alcohol and cannabis, and even demonstrate higher accounted variance for an initial psychometric analysis (Martens et al., 2005; Pedersen et al., 2016).The PSPS had excellent convergent validity with these validated PBS measures. Overall, these preliminary analyses suggest that the two-factor PSPS could assess protective strategies for psychedelic use.

Findings from this study offer numerous important contributions to the study of psychedelics. The principal contribution is the development of a validated assessment tool for psychedelic protective strategies. Presently, we see PBS scales for other substances commonly cited and employed, but no such tool exists for psychedelics (Martens et al., 2005; Pedersen et al., 2016). A clear need exists for creating such an instrument, and this developed scale offers an important first step for understanding this construct. Developing psychometric research on the preparation for psychedelic experiences further illustrates the importance of harm reduction strategies in the context of psychedelic use (McAlpine et al., 2023). Given our own initial preliminary analysis, further psychometric examination including confirmatory factor analyses and invariance testing are critical to the development and dissemination of this scale. Nevertheless, the present PSPS scale provides an efficient translation of intuitive and traditional harm reduction strategies into a new form that is standardized and can now be more easily shared.

The developed PSPS scale has important implications for future research. This initial analysis demonstrated a two-factor solution for long- and short-term strategies was most appropriate for the current data. This preliminary finding potentially suggests that individuals are engaging in a variety of harm mitigating strategies, that includes immediate and more advanced preparation. Strategies also spanned diverse domains, including social and environmental context, health, and affect. This corroborates prior work examining harm mitigation behaviors (Hupli et al., 2019; Lancelotta and Davis, 2020a; Lea et al., 2020; Ruane, 2017). Future work might reveal additional potential underlying constructs for psychedelic-specific PBS. Additionally, while our study demonstrated the consistent associations between both factors and psychedelic use, examinations of other relevant constructs might uncover unique associations between the factors and those constructs, as demonstrated with short forms of the PBSM (Jordan et al., 2022; Mian et al., 2021). Notably, the PSPS had a moderate positive relation to lifetime psychedelic use. This contrasts with both the PBSM and PBSS, which both had negative associations with cannabis use and psychedelic use respectively (Martens et al., 2005; Pedersen et al., 2016). This deviation does not minimize the utility of the PSPS; in fact, it might underscore the unique behaviors and effects associated with psychedelic use that differ from other substances (Carhart-Harris et al., 2018; Haijen et al., 2018). Potentially, psychedelic protective strategies might develop in parallel to experience and repeated use. Further, using psychedelics more frequently could simply provide more opportunities to use protective strategies. Future work can replicate and extend these findings to better understand the complex associations between use and protective strategies. Given the diversity of psychedelic substances (e.g., classic psychedelics, entactogenic psychedelics, dissociative psychedelics), some strategies might prove more relevant for some psychedelics than others. A safe environment, for example, likely has universal application. In contrast, abundant water might minimize consequences more for substances with a longer duration or stereotypically dehydrating, emetic, or stimulating effects. Only very large replication samples could identify drug-specific links of some protective behaviors with problems. Nevertheless, the specificity of protective behaviors could help focus efforts to prevent harm.

Little work has examined harm reduction strategies in relation to use outcomes for psychedelics. Our work answers initial questions about the associations between protective strategies and outcomes such as use, but future work can examine other factors relevant to psychedelics such as negative consequences and quality of mystical experiences (MacLean et al., 2012). Future work might use this assessment tool to better understand the mechanisms between harm reduction and use-related outcomes. This work generally highlights the need for more psychometrically validated assessment tools for psychedelics, including for a psychedelics-related problems scale. Additionally, this scale can elucidate the prevalence of harm reduction strategies among different contexts and identify commonly endorsed strategies in different groups of people, particularly those who might be vulnerable to problems associated with use. Information about psychedelic protective strategies can inform interventions to mitigate risky use and unintended consequences, as seen with other PBS scales (Bravo et al., 2017; Pearson et al., 2017; Pedersen et al., 2017).

Finally, the PSPS can potentially be meaningful in clinical settings. Individuals using psychedelics can benefit by assessing their own harm reduction strategies with the PSPS. Such individuals who self-dose for therapeutic or recreational reasons might mitigate potential harm through such assessments. Additionally, as the effectiveness of psychedelic-assisted therapy continues to be investigated, clinicians might benefit from resources, such as manuals for these interventions, that could incorporate protective strategies into their protocols.

The present study is not without limitations. As with any novel scale development, further rigorous psychometric testing is essential to improving the validity of the measure. The final scale included 32 of the original 37 items developed for the analysis. Iterative analyses, including confirmatory factor analysis and invariance testing might reveal other dimensions or lead to the removal of items, as demonstrated in other studies developing PBS scale (Martens et al., 2007; Mian et al., 2021; Pedersen et al., 2017). This replication will be critical to validating the scale. The scale’s validation was also limited to our sample; most participants identified as White. While one strength of our study is drawing from a community sample, additional testing can provide an opportunity to examine the scale’s performance in the context of a more diverse sample and clinical samples, and further investigate the role of context and motivation for use in employing particular kinds of protective strategies (Carhart-Harris et al., 2018). In addition, our sample reported lifetime use of multiple different kinds of psychedelics, and with our present sample size, it was not possible to examine associations between specific strategies and specific psychedelics. Given the diversity of psychedelics and subsequent effects, future work would certainly benefit from examining these associations. Nevertheless, our recruitment of a sample using different psychedelics is consistent with other scale development work on psychedelics (Peill et al., 2022; Roseman et al., 2019; Wolff et al., 2022).

Finally, our study was able to assess and demonstrate strong convergent validity between the PSPS with other PBS scales, though future work should examine the scale and its factors in relation to other relevant constructs, such as negative consequences and other factors related to psychedelic experiences. This study only assessed cannabis and alcohol use as both have validated PBS scales, but future work would benefit from examining other substance use in relation to psychedelic use and harm reduction strategies.

## Conclusion

The renewed interest in psychedelics calls for the inclusion of harm reduction strategies to help individuals use them safely and avoid unintended consequences, yet no validated scale for this construct currently exists. Our preliminary psychometric study developing a PSPS offers an important first step in addressing this critical gap. The PSPS demonstrates promising psychometric properties at this early stage, and we anticipate future work will further validate the scale for diverse samples across research and clinical settings. We encourage scholars and clinicians alike to consider harm reduction and continue the development and dissemination of protective strategies that can empower individuals to use psychedelics safely and effectively.
